# A Microbial Endocrinology-Designed Discovery Platform to Identify Histamine-Degrading Probiotics: Proof of Concept in Poultry

**DOI:** 10.3390/microorganisms13040751

**Published:** 2025-03-26

**Authors:** Mark Lyte, Karrie Daniels

**Affiliations:** Department of Veterinary Microbiology and Preventive Medicine, College of Veterinary Medicine, Iowa State University, Ames, IA 50011, USA; karriew@iastate.edu

**Keywords:** biogenic amine, *Brevibacterium* spp., growth performance, histamine, Microbial Endocrinology, poultry, probiotic

## Abstract

Histamine is a biogenic amine found across the phylogenetic spectrum, from plants to fish to animals. In farm animal production, the host’s production of histamine within the intestinal tract serves as a neurotransmitter, facilitating communication from the gut to the brain. Histamine functions additionally as a “bridging” chemical between the immune and nervous systems as it facilitates nervous system modulation of host immune response, thereby playing a critical role in host defense within the gut. Increased histamine levels within the gut, whether originating from food-borne sources or produced in situ, can lead to immune dysregulation and consequent physiological harm. As such, control of histamine within the gut can improve overall gut health across a broad range of species. In the present study, we utilized a Microbial Endocrinology-based approach as a platform technology to enable the discovery of unique histamine-degrading bacteria within the gut microbiota. Broiler chickens were fed, or not, a low or high histamine-supplemented diet from one day of age to up to 42 days in order to encourage the increased abundance of putative histamine-degrading bacteria. Intestinal contents were employed in a discovery protocol that involved repeated isolation rounds utilizing a histamine-supplemented minimal medium. We herein report the discovery that the genus *Brevibacterium* are capable of up to 100% degradation of histamine in vitro. Feeding experiments utilizing one of the identified *Brevibacterium* spp., a *B. sediminis* isolate, demonstrated that it reduced the amount of histamine in the gut of broilers fed a histamine-containing diet and enabled an improvement in growth as compared to non-*B. sediminis*-supplemented animals. As such, this study demonstrates the usefulness of a Microbial Endocrinology-based approach for the discovery of bacteria that may serve as potential probiotic candidates for the control of neurochemical-mediated interactions within the host, thereby improving host health.

## 1. Introduction

As a biogenic amine, histamine is found widely dispersed throughout the plant and animal kingdoms. Within the gut of mammals, external and internal sources of histamine contribute to overall levels within the gastrointestinal tract. Dietary inputs, such as histamine from plants or as part of an animal-based diet, can provide large amounts of histamine [1,2]. Further, histamine-producing bacteria may also be consumed as part of the diet [3]. Within the gut, enterochromaffin-like cells and specific neurons in the enteric nervous system further contribute to the overall concentration of histamine [4,5,6]. Additionally, bacteria present within the gut, notably *Staphylococcus* spp., *Clostridium* spp., and *Proteus* spp., can produce histamine from histidine derived from protein metabolism [3]. Within the gut, histamine plays a crucial role in a number of physiological processes, ranging from the mediation of smooth muscle contraction and gastric acid section to the modulation of immune responsiveness [7,8]. Indeed, histamine is an important mediator between intestinal mast cells and the enteric nervous system [9]. Histamine also serves as a chemical mediator, enabling gut–brain communication that can influence brain function and ultimately behavior [10]. While these aforementioned roles of histamine in physiology are crucial to the maintenance of homeostasis, excessive histamine within the gut can result in a number of physiological consequences affecting gastrointestinal, cardiovascular, and neurological systems. For example, in humans with histamine intolerance, excessive levels of histamine can result in life-threatening conditions such as anaphylaxis [11,12].

The need to control histamine within the gut has been recognized for decades. Possible means have included dietary manipulation [13] and the use of oral histaminase, also referred to as diamine oxidase, preparations prepared from plants [14] or porcine kidneys [15]. Although these have met with some success primarily in humans [14], there is still a critical need for additional modalities not only for the treatment of histamine intolerance in humans but also in farm production animals, where histamine is also of primary concern due to its deleterious effects on gut health and ultimately growth performance [1,16,17]. In poultry, for example, diets containing spoiled animal by-products, such as spoiled fish meal, can lead to excessive levels of histamine within the gut, adversely affecting broiler gut health and dysregulation of the immune system [18,19]. The use of treatment modalities that have met with limited success in humans, such as oral histaminase [14], is not feasible in farm production animals due to their exceedingly high cost which can range into hundreds of dollars per person per month. A possible avenue by which farm production animals, and of obvious benefit to humans as well, could be explored is the use of histamine-degrading probiotics. While there have been some reports demonstrating possible beneficial effects, there have been few regarding their use in the animal industry [20,21].

The present manuscript describes a new method to achieve the goal of controlling gut histamine in farm production animals, with an obvious translation to humans. We have chosen poultry as the animal species from which to obtain proof-of-concept data, as this species offers a number of distinct advantages, particularly concerning the modeling and role of histamine in gut inflammation [22]. This new research avenue described in the present report involves the use of Microbial Endocrinology, which represents the union of the fields of neurophysiology and microbiology, as a conceptual, evidence-based framework to identify and select normal constituents of the animal’s microbiota to expand out and employ as a histamine-degrading probiotic. The theoretical underpinnings discussing the rationale by which the use of Microbial Endocrinology can serve as an evidence-based framework to discover or design function-driven probiotics, which operate due to their ability to modulate neurochemistry within the host, have been previously discussed [23].

## 2. Materials and Methods

### 2.1. Ethical Statement

The experiments were approved and conducted in accordance with guidelines set by the Iowa State University Animal Care and Use Committee. The trials were conducted at the Iowa State University College of Veterinary Medicine animal facilities in Ames, IA, USA.

### 2.2. Animals and Histamine Feeding Trials

**Figure 1:**
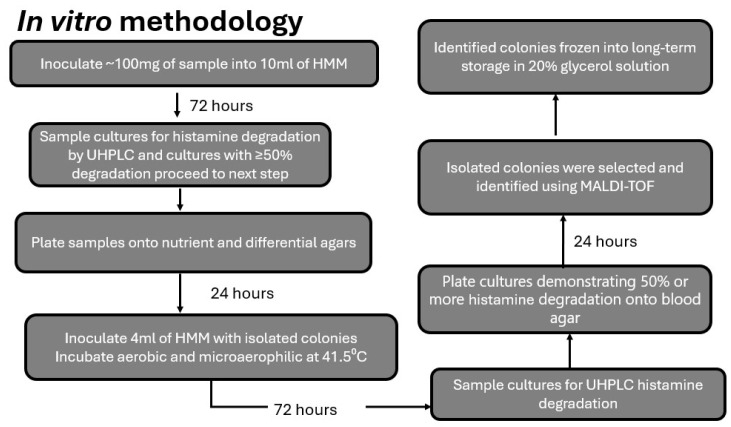
Cecal contents (~100 mg) from broilers fed a control, low (10 mg histamine per kg of feed), moderate (30 mg histamine per kg of feed), or high (100 mg histamine per kg of feed) diet were employed in the protocol shown as fully described in the Materials and Methods.

**Figure 2:**
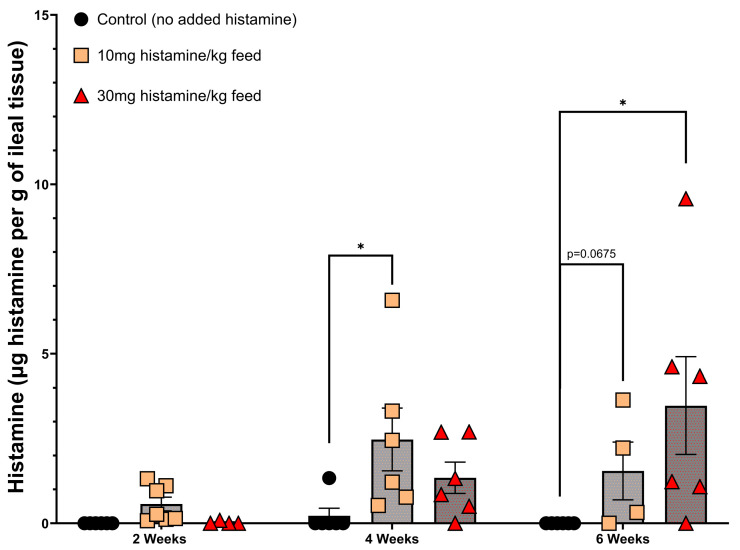
Histamine concentrations for ileal tissue from broiler chickens fed a control, low (10 mg histamine per kg of feed), or high (30 mg histamine per kg of feed) diet and sacrificed at the length of time on the diet as shown. Data were screened for outliers using the Grubbs test and then analyzed with outliers removed using two-way ANOVA followed by Fisher’s LSD post hoc test. Each symbol represents an individual chicken (n = 4–7 chickens per group). All data are presented as mean ± SEM. Asterisk indicates statistical significance at *p* < 0.05.

Trials employed male broiler chickens (male Cornish Rock 1-day-old chickens; Welp Hatchery, Bancroft, IA, USA). It is worth noting that although in the present study, we utilized the Cornish Rock breed, the present findings are also likely applicable to Cobb and Ross commercial broiler breeds. This is because a variety of chicken breeds have, for decades, been studied for intestinal histamine and the role of the microbiota in modulating tissue concentrations of histamine [24,25]. Upon receipt, chicks were randomly divided into experimental treatment groups of n = 15–17 chicks per group and raised to 14 or 42 days of age, depending on the length of the individual trial, as shown in the figures. Animals were sacrificed and cecal contents were harvested and then employed in the histamine-degrading probiotic discovery protocol, as shown in Figure 1. For the trial involving the control vs. low histamine vs. medium histamine-containing diets (Figure 2), n = 4–7 chickens from the initial starting group of 15–17 chickens were sacrificed at two-week intervals (i.e., 2 weeks, 4 weeks, and 6 weeks of age) over the 42-day trial. All chickens were housed in floor pens with fresh litter. Crumbled poultry diet (Purina Start and Grow Non-Medicated Chick Feed, #3004800303, Purina Mills, Riverside, MO, USA) was supplemented, or not, with histamine (histamine dihydrochloride; CAS 56-92-8, TCI Chemicals, Portland, OR, USA) by thoroughly mixing the crumbled poultry diet with histamine to generate low (10 mg histamine/kg of feed), moderate (30 mg histamine/kg of feed), or high (100 mg histamine/kg of feed) histamine or non-histamine containing feeds starting at Day 1 age and throughout the 42-day feeding period. 

Diets, either supplemented or not with histamine, were fed using poultry-specific feeders ad libitum. Chickens were provided ad libitum access to water throughout the entire study period. The levels of histamine employed did not produce any adverse histopathological effects, as confirmed by postmortem examination of selected animals by an avian pathologist blinded to the groups. Chickens were weighed prior to sacrifice.

### 2.3. Quantitative Measurement of Histamine Levels

The quantitative determination of histamine was performed by ultra-high-performance liquid chromatography (UHPLC) with UV-VIS detection as previously reported [24].

### 2.4. Collection of Biological Samples

Chickens were sacrificed using carbon dioxide euthanasia. Immediately following euthanasia, the entire gastrointestinal tract was rapidly removed intact. The colon was then severed at the point of the ceca-colon junction and then halved laterally to produce proximal and distal colon sections. The bifurcated ceca were removed and approximately 100 mg of cecal content was immediately collected and placed into a tube for culturing and identification of histamine-degrading bacteria as described below.

### 2.5. Isolation of Histamine-Degrading Bacteria from Histamine Fed Broilers

As shown in the flow diagram (Figure 1), the isolation of histamine-degrading bacteria followed a stepwise protocol. Following sacrifice, approximately 100 mg of cecal content was removed from each of the left and right ceca and mixed before transferring to a tube containing histamine minimal medium (HMM), which was then thoroughly mixed on a vortex. The composition of HMM is based on a previous report that utilized it for the isolation of histamine-degrading bacteria from cheese [26]. In brief, HMM consisted of 77.6 mM potassium phosphate dibasic (Sigma-Aldrich, CAS-7758-11-3, St. Louis, MO, USA), 15.1 mM ammonium sulfate (Fisher Scientific, CAS-7783-20-2, Waltham, MA, USA), 1 mM magnesium sulfate heptahydrate (Sigma, CAS-10034-99-8), 24.6 µM biotin (TCI, CAS-58-85-5), 71.2 µM thiamine hydrochloride (Sigma, CAS-67-03-8), 12.6 µM calcium D-pantothenate (TCI, CAS-137-08-6), and 10 mM histamine dihydrochloride with a final pH of 6.5. Due to the composition of HMM, only bacteria that were capable of utilizing histamine as an energy source would proliferate. Following 72 h static culture at 41.5 °C, cultures were sampled for percentage of histamine degradation. If the percentage of degradation was greater than 50% of the concentration present at the start of the culture, 100 µL aliquots were removed, serially diluted, and then plated onto nutrient and differential agars. The differential agars consisted of Columbia Nalidixic Acid, MacConkey, Eosin methylene blue, de Man–Rogosa–Sharpe (MRS), 5% sheep blood, and 1/3 strength Brain Heart Infusion. Following overnight growth, isolated colonies were picked from plates and then dispersed into fresh HMM and incubated for 72 h. Aliquots from the HMM tubes were then removed and tested for percent histamine degradation. Tubes that evidenced greater than 50% histamine degradation were then plated onto 5% sheep blood agar. Following overnight incubation, colonies from plates that showed only a single, uniform morphology, were picked and identified using mass spectrometry-based matrix-assisted laser desorption/ionization (microflex MALDI-TOF system, Bruker, Billerica, MA, USA). Following MALDI-TOF identification, cultures were then placed into −84 °C storage using a 20% glycerol stock freezing solution.

### 2.6. Brevibacterium Culture Stocks

*Brevibacterium* spp. isolates were obtained from a national culture collection repository (USDA ARS Culture Collection (NRRL), Peoria, IL, USA), as well as from an Iowa State University cheese researcher (kind donation of Dr. Stephan Schmitz-Esser, Department of Animal Sciences, Ames, IA, USA).

### 2.7. Incorporation of Histamine-Degrading Brevibacterium Probiotic into Feed

Candidate *Brevibacterium* spp. isolates were incorporated into crumbled poultry feed using a freeze-drying preparation method prior to being added to the feed. The selected Brevibacterium isolates were first expanded by overnight growth at 41 °C in 1 L Erlenmeyer flasks placed in an incubator shaker (Eppendorf Innova 44, Hamburg, Germany) with a rotational speed of 140 rpm. Following overnight growth, the flask contents were spun at 4000 rpm for 10 min, and the supernatant was discarded. The bacterial pellet was then resuspended in 10 mL of Reagent 18 (GFS Chemicals, Columbus, OH, USA), which served as a lyoprotectant during the subsequent freeze-drying.

For the lyophilization method, the bacterial suspension was applied to 4 kg of crumbled chicken feed and mixed by hand. The feed mixture was then transferred to a Harvest Right Freeze Dryer (Harvest Right, Salt Lake City, UT, USA) and was lyophilized overnight before transferring to a storage container. Following completion of the freeze-drying, the bacteria were then incorporated into the feed at a concentration of 5 × 10^9^ to 1 × 10^10^ per kg of feed. The viability of bacteria in feed was assessed by sampling feed at various times following addition into feeders.

### 2.8. Statistical Analysis

Graphing and statistical analyses utilized the GraphPad Prism software (version 10, GraphPad, La Jolla, CA, USA). Data were analyzed, where appropriate, using two-way ANOVA followed by Fisher’s LSD post hoc test, or analyzed by a two-tailed *t*-test, and a *p*-value < 0.05 was considered statistically significant.

## 3. Results

### 3.1. Histamine Feeding Trial and Identification of Histamine-Degrading Bacteria

As shown in Figure 2, feeding of histamine resulted in an increase in levels of histamine within the ileal tissue as compared to the control (non-histamine-fed) chickens. Following sacrifice at the end of the 6-week trial, cecal contents were removed and employed in the discovery protocol, as shown in Figure 1.

**Figure 3:**
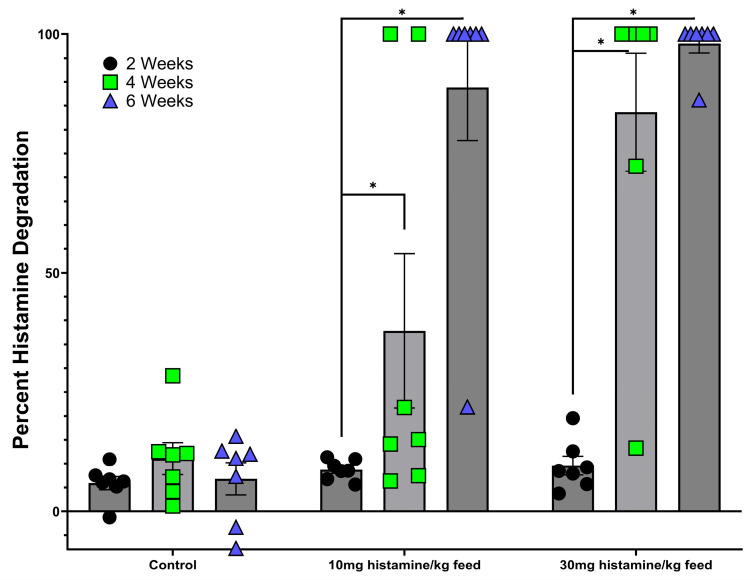
Percent of histamine degradation for cecal cultures from broilers fed a control (no histamine added), low (10 mg histamine per kg of feed), or moderate (30 mg histamine per kg of feed) diets and employed in the protocol shown in Figure 1. Results are for the first step of the protocol, in which cultures were sampled after 72 h incubation of the cecal material in HMM. If cultures at this first stage showed 50% or greater histamine degradation, they were then employed in subsequent steps of the protocol. Data were analyzed using two-way ANOVA followed by Fisher’s LSD post hoc test. All data are presented as mean ± SEM. Asterisk indicates statistical significance at *p* < 0.05.

As shown in Figure 3, cecal cultures from control, non-histamine-fed birds exhibited a minimal ability to degrade histamine in culture. Over the 6-week trial period, the low histamine-degrading capacity in control birds did not change. Interestingly, in the groups fed a histamine-containing diet, the ability of cecal cultures to degrade histamine increased over the course of the trial. As shown in Figure 3, by the end of the trial nearly 100% of all the cecal cultures from the low histamine-fed (10 mg histamine/kg feed) group and 100% of all the cecal cultures from the moderate histamine-fed (30 mg histamine/kg feed) group were capable of complete degradation of histamine in the HMM test medium. Results from the MALDI identification of various isolates demonstrated the presence of a number of genera that were capable of histamine degradation. Notably, a majority of isolates were identified as belonging to the genus *Brevibacterium*. Other genera isolated included *Pseudomonas* and *Enterococcus*. As discussed in the Discussion Section, due to the unexpected finding of *Brevibacterium* spp., and the non-suitability of the other identified genera as potential probiotics due to the presence of antibiotic-resistant genes, we decided to further explore the potential of the *Brevibacterium* spp. to be scaled up as a histamine-degrading feed additive.

### 3.2. Evaluation of Brevibacterium spp. Bacteria for Histamine-Degrading Capability

**Figure 4:**
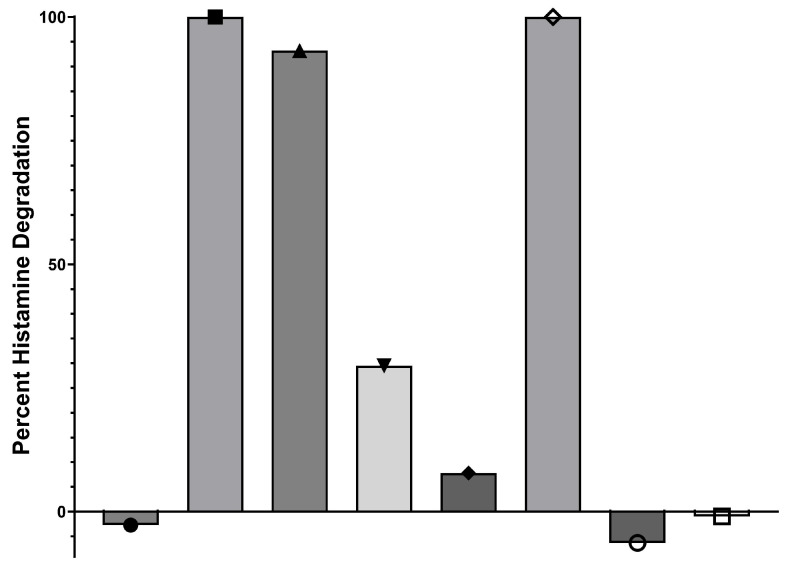
The ability of *Brevibacterium* spp. isolates obtained from stock culture collections to degrade histamine in HMM. ●, *B. iodinum* (ML1189); ■, *B. sediminis* (ML1197); ▲, *B. sediminis* (ML1205); ▼, *B. epidermidis* (ML1268); ♦, *B. aureum* (ML1199); ◊, *B. sediminis* (ML1203); ○, *Brevibacterium* spp. (ML1192); □, *Brevibacterium* spp. (ML1293).

As per the isolation protocol, isolates were frozen in nutrient broth supplemented with 20% glycerol as a cryoprotectant at −84 °C. Isolates were removed at a later date from −84 °C storage and placed into culture to confirm that their ability to degrade histamine remained intact. Experiments utilizing the *Brevibacterium* spp. isolates proved variable in their ability to degrade histamine following storage at −84 °C. The reasons for this development are unclear but are not without precedent, as microbiological cultures can often lose certain physiological and metabolic capabilities following long-term cold storage, whether due to the selection of a subpopulation of cells capable of withstanding the freezing and thawing process or some other yet-to-be-identified mechanism. Regardless, to further pursue the examination of *Brevibacterium* spp. as potential histamine-degrading probiotics, we employed isolates from a national culture collection (USDA ARS Culture Collection (NRRL)) or from an Iowa State University researcher who had published on the role of *Brevibacterium* spp. in the degradation of histamine in cheese rind (kind donation of Dr. Stephan Schmitz-Esser, Department of Animal Science [26]). As shown in Figure 4, a number of the *Brevibacterium* spp. isolates were capable of 100% degradation of histamine in HMM over a 72 h incubation period. Non-poultry isolates did in fact display robust ability to degrade histamine but only at lower temperatures.

### 3.3. Evaluation of B. sediminis as a Probiotic Feed Additive to Control Gut Histamine

**Figure 5:**
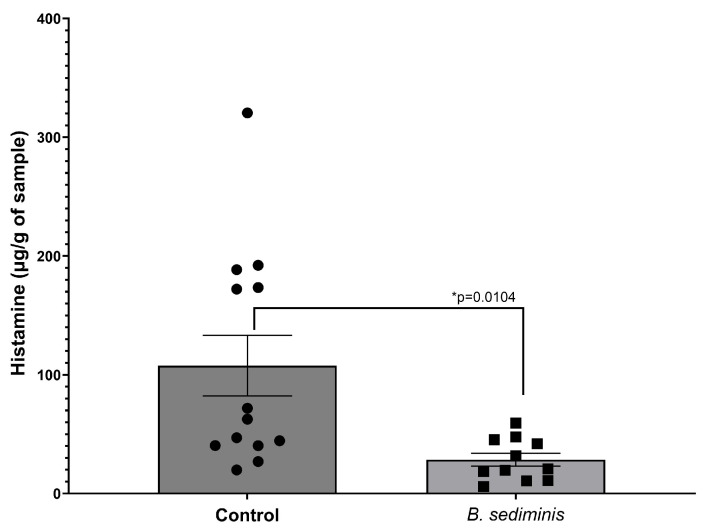
Histamine concentrations in cecal content obtained from broiler chickens fed a high (100 mg histamine per kg of feed) diet, supplemented or not with the *B. sediminis* probiotic at a concentration of 5 × 10^9^ to 1 × 10^10^ CFU per kg of feed for 14 days. Each symbol represents an individual animal. The results shown are the mean ± SEM. Data were analyzed using a two-tailed *t*-test.

Based on the results shown in Figure 4, *B. sediminis* ML1197 was selected for evaluation as a potential probiotic for use in poultry feed. As detailed in Materials and Methods, a freeze-drying method was employed to prepare *B. sediminis* prior to its incorporation into the poultry feed. As shown in Figure 5, animals fed a histamine-supplemented diet (100 mg histamine per kg of feed), which did not contain the *B. sediminis* probiotic, for 14 days demonstrated significant levels of histamine in the cecal contents.

**Figure 6:**
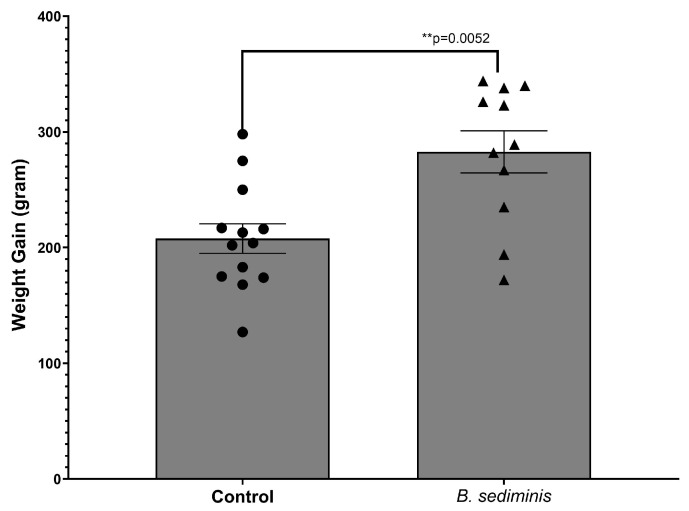
Amount of weight gain of broiler chickens fed a high (100 mg histamine per kg of feed) diet, supplemented or not with the *B. sediminis* probiotic at a concentration of 5 × 10^9^ to 1 × 10^10^ CFU per kg of body weight for 14 days. Weight gain was determined as the difference between the start (one day following hatch) and finish (time of sacrifice) weights for each individually identifier-tagged bird during the 14-day trial). Each symbol represents an individual animal. The results shown are the mean ± SEM. Data were analyzed using a two-tailed *t*-test.

Animals fed the *B. sediminis* probiotic at a concentration of 5 × 10^9^ to 1 × 10^10^ CFU per kg of feed, in conjunction with the histamine-supplemented diet, demonstrated a significant reduction in cecal content histamine as compared to the non-probiotic control. Further, animals that were fed the histamine-containing diet supplemented with the *B. sediminis* probiotic also displayed greater weight gain by the end of the trial as compared to non-probiotic-fed, histamine-diet-fed control chickens (Figure 6).

## 4. Discussion

Histamine, which is derived from the decarboxylation of the amino acid L-histidine by histidine decarboxylase, is a biogenic amine widely dispersed throughout nature and whose pleiotropic effects in the host are well recognized. Its role as a signaling molecule in a number of physiological systems, ranging from immune to neurological to digestive, has been well documented for decades (for review see [8]). These physiological effects include, but are not limited to, roles in immunological function, such as a mediator of inflammatory responses, especially within the gut to its role as a neurotransmitter in the brain-regulating behavior [27,28,29]. Not unexpectedly, the concentration of histamine— whether produced by the host or consumed as part of the diet, as with any biogenic amine—is crucial to its ability to function in the maintenance of normal homeostasis in the organisms, whether they area food production animals, fish, or humans [2,6,10]. Increases in histamine levels above what which is needed for its normal regulation of host physiology can result in a spectrum of adverse consequences. For example, within the gut, excessive histamine disrupts mucosal barrier integrity, which has been shown to be a critical factor in mediating the development of inflammation [5,29]. From a behavioral perspective, increased histamine can lead to increased anxiety and aggression [28]. In humans, an excess of histamine leads to a condition that has been termed histamine intolerance and is characterized by digestive issues, such as abdominal pain and diarrhea, headaches, fatigue, shortness of breath, and low blood pressure [11]. Consumption of foods containing excess histamine or even the use of medications such as antibiotics, which can result in the release of histamine in the body, can result in severe anaphylaxis that may lead to death [30,31,32]. However, these negative consequences of excess histamine are not solely restricted to humans. The feed used for animal food production, such as in the poultry industry, can use fish meal as an inexpensive source of protein and other nutrients [19,33]. As fish naturally harbor histamine-producing microorganisms, contamination of the industrial-scale production of fish meal with these histamine-producing microorganisms, such as *Morganella morganii* [34], results in a product with a high histamine level that is then incorporated into the final animal feed product. In poultry, the consumption of such histamine-rich feed has been demonstrated to result in gizzard erosion and consequent reduction in overall growth rates [19,35].

It should, therefore, be apparent that the control of histamine, in particular avoidance of high histamine levels in the host, is of primary importance to health and behavior. While pharmacological preventative and treatment measures have been employed for decades [36], the use of a microbial-based approach, and, in particular, the use of bacteria (probiotics), has been far less intensively investigated. Studies that do examine the use of putative probiotics mainly center on the use of histamine-reducing bacteria or bacterial extracts to reduce the level of histamine in foods known to contain high levels before consumption. For example, wine is known to often contain high levels of histamine that may induce allergic reactions following consumption [37]. Callejon et al. [38] have shown that enzymatic extracts obtained from lactic acid bacteria can be used to reduce the concentration levels of histamine. Analogously, the isolation of potential probiotics exhibiting histamine-degrading capabilities has been shown in two strains of *Lactobacillus paracasei* isolated from artisanal cheese [39]. It should be noted that not all probiotic-related publications concerning histamine have highlighted the negative consequences of histamine. The use of the histamine-secreting probiotic *Lactobacillus reuteri* in combination with a histidine-containing diet has been shown to suppress the development of gut inflammation in a trinitrobenzene sulfonic acid (TNBS)-induced mouse colitis model [40]. The presence of a bacterial histidine decarboxylase gene to convert the dietary-provided histidine to histamine was shown to be an absolute requirement for the observed ability of the probiotic to suppress inflammation.

In order to achieve this goal, the present report describes the use of a Microbial Endocrinology-based discovery platform to isolate histamine-degrading bacteria present in the gut that could then be validated in vitro and subsequently utilized as a probiotic to reduce histamine within the gut. We chose to employ poultry, specifically broiler chickens, as our proof-of-concept model system. As a tractable model testing system, poultry offers a number of unique benefits. First, the deleterious effects of excess histamine on poultry health have been well documented. The first reports of histamine-induced pathology in poultry concerned the consumption of spoiled fish meal that was part of the feed [19]. Tucker et al. demonstrated that the spoiled fish meal component of the diet had been contaminated by bacteria, most likely originating from the fish gut, that resulted in the production of high levels of histamine. Feeding on this diet resulted in a number of pathological changes, including gizzard erosion and necrotic enteritis that adversely affected growth performance and resulted in increased mortality [19]. Secondly, due to their size, poultry offers the advantage of obtaining larger tissue and gut content sample sizes than can be obtained in other animal models such as those, which utilize mice. Thirdly, poultry has been extensively used as a model for the role of different nutritive elements, including pre and probiotics, in the maintenance of gut health. For example, Obianwuna et al. [41] utilized a broiler chicken model to evaluate the ability of phytobiotics to mitigate the effects of oxidative stress on intestinal health. For a number of decades, poultry has been employed to evaluate the utility of probiotics in overall gut health, including the prevention of infectious disease [42,43].

The use of a histamine-enriched diet to encourage the proliferation of histamine-degrading bacteria within the microbiota was an essential key to the Microbial Endocrinology platform as its purpose was to exploit the dynamics underlying the basis that neurochemicals, such as histamine, serve as a common element uniting the seemingly disparate fields of microbiology and neurobiology [44,45]. As shown in Figure 2, feeding of two different levels of a histamine-containing diet resulted in the emergence of bacteria within the cecum that were capable of nearly 100% degradation of a high (10 mM) concentration of histamine in the test HMM (Figure 3). Interestingly, no such histamine-degrading capacity was observed in animals fed a control, non-histamine-containing diet (Figure 2). Further, the continual feeding of the histamine-containing diet over time resulted in greater capacity of the cecal contents to degrade histamine in the in vitro testing medium (Figure 3). Following the protocol shown in Figure 3, we identified a number of genera that showed an ability to degrade histamine. Not unexpectedly, *Pseudomonas aeruginosa* was identified in the isolation procedure. Although the ability of *Pseudomonas* spp. to degrade histamine is well recognized [46], it would not be considered a suitable probiotic candidate and thus was not further pursued. However, one of the gut bacterial genera consistently identified as a highly efficient histamine degrader was that of *Brevibacterium* (Figure 3). Repeated efforts at freezing and recovery of the isolates, unfortunately, did not result in consistently viable cells that could be further expanded for use as a probiotic in planned feeding trials. As such, we reverted to the use of *Brevibacterium* spp. that had been isolated from the fecal matter of broiler chickens or obtained from the USDA national repository. As shown in Figure 3, a number of these strains, in particular *B. sediminis*, were capable of 100% degradation of 10 mM histamine within 72 h at 41.5 °C. Based upon the results shown in Figure 3, we chose to expand *B. sediminis* to utilize in subsequent feeding trials.

The discovery that *Brevibacterium* spp. within the poultry gut was responsible for histamine degradation was, in retrospect, not surprising. *Brevibacterium* constitutes a genus of Gram-positive, non-motile, catalase-positive bacteria with low pathogenic potential [47,48]. *Brevibacterium* spp. have been used for hundreds of years in the manufacture of cheese and, as such, form an integral part of the microbiota that comprises the rind. While their role in providing flavor and aroma to the various cheese varieties was well-recognized, it is their ability to degrade histamine produced by bacteria during manufacture that is crucial to the safety of the food product [49]. Recently, the mechanism by which *Brevibacterium* benefits cheese manufacture has been elucidated. Anast et al. [26] demonstrated that members of the *Brevibacterium* genus, such as *B. aurantiacum*, which are used in the manufacture of Austrian cheese, possess metabolic pathways for histamine degradation. Interestingly, the presence of *Brevibacterium* spp. within the poultry gut has been known for decades [50]. However, the putative function of *Brevibacterium* spp. within the poultry gut has been unknown. In fact, due to the relative absence of *Brevibacterium* spp. in the larger environment, its presence has been utilized as a marker for water pollution by fecal matter downstream from poultry production facilities [51]. As such, this report also represents the first known function that can be ascribed to *Brevibacterium* spp. in poultry.

As histamine is a potent modulator of immunity [27,52], having less histamine present within the mucosal tissue, which contains the immune cells, such as macrophages, T lymphocytes, and eosinophils, as well as dendritic cells, responsible for the pathogenesis of the inflammatory state is desirable [5,29]. Critically, it is also noteworthy that excess histamine is recognized to be an indicator of reduced growth performance in animal farm production species other than poultry. For example, a recent report by Ramsay et al. [16] evaluated over 576 metabolites in piglets to identify potential metabolite markers that could be used as predictors of animal performance. They reported that only peripheral histamine levels could serve as a reliable marker to predict subsequent growth performance as well as identify which animals would be slow-growing and which would attain normal growth levels. Thus, the results presented in Figure 6, as well as overall in this report, provide proof-of-concept data that demonstrate that the regulation of gut histamine through the use of a Microbial Endocrinology-designed probiotic targeting a neurochemical recognized to regulate the interface between the immune and nervous systems can help regulate homeostasis, as reflected in increased growth performance.

## 5. Conclusions

Given the results presented herein, the present study demonstrates that a Microbial Endocrinology-based approach to the mechanistic design of probiotics can result in a probiotic that functions through the utilization of a neurochemical to benefit animal health and, by extension, human health [23,45]. Microbial Endocrinology represents the union of two seemingly disparate fields: microbiology and neurobiology [23,45,53]. The ability of microorganisms, ranging from bacteria to yeasts, has been known for decades to possess the ability to produce and recognize neurochemicals that otherwise have been primarily associated with mammals. Included among these neurochemicals is histamine, which is, in fact, produced by a wide variety of microorganisms [3,52]. The ability of histamine to impact physiological function extends well beyond that of its effects within the gut. For example, work performed by Barcik et al. [54] demonstrated that histamine produced in the gut could influence immune responses within the lung. Thus, controlling histamine levels, especially through the utilization of a probiotic, offers a new preventative/treatment option that is applicable in farm production animals as well as humans. Additionally, the results presented in this report suggest that targeting neurochemicals that serve as an interface between microbiology and neurobiology (i.e., Microbial Endocrinology), represents a viable platform technology for the development of a new generation of targeted, mechanistically designed, probiotics.

## 6. Patents

As the Assignee, The Iowa State University Research Foundation has filed patents surrounding the discovery and utilization of the histamine-producing probiotic described in this report.

## Data Availability

The original contributions presented in this study are included in the article. Further inquiries can be directed to the corresponding author.

## Abbreviations

The following abbreviations are used in this manuscript:

HMM	Histamine minimal medium
UHPLC	Ultra-high performance liquid chromatography
